# Volumes of brain structures in captive wild-type and laboratory rats: 7T magnetic resonance *in vivo* automatic atlas-based study

**DOI:** 10.1371/journal.pone.0215348

**Published:** 2019-04-11

**Authors:** Marlena Welniak–Kaminska, Michal Fiedorowicz, Jaroslaw Orzel, Piotr Bogorodzki, Klaudia Modlinska, Rafal Stryjek, Anna Chrzanowska, Wojciech Pisula, Pawel Grieb

**Affiliations:** 1 Department of Experimental Pharmacology, Mossakowski Medical Research Centre, Polish Academy of Sciences, Warsaw, Poland; 2 Small Animal Magnetic Resonance Imaging Laboratory, Mossakowski Medical Research Centre, Polish Academy of Sciences, Warsaw, Poland; 3 Institute of Radioelectronics and Multimedia Technology, Faculty of Electronics and Information Technology, Warsaw University of Technology, Warsaw, Poland; 4 Institute of Psychology, Polish Academy of Sciences, Warsaw, Poland; King's College London, UNITED KINGDOM

## Abstract

Selective breeding of laboratory rats resulted in changes of their behavior. Concomitantly, the albino strains developed vision related pathologies. These alterations certainly occurred on the background of modifications in brain morphology. The aim of the study was to assess and compare volumes of major structures in brains of wild-captive, laboratory albino and laboratory pigmented rats. High resolution T2-weighted images of brains of adult male Warsaw Wild Captive Pisula-Stryjek rats (WWCPS, a model of wild type), laboratory pigmented (Brown Norway strain, BN) and albino rats (Wistar strain, WI) were obtained with a 7T small animal-dedicated magnetic resonance tomograph. Volume quantification of whole brains and 50 brain structures within each brain were performed with the digital Schwarz rat brain atlas and a custom-made MATLAB/SPM8 scripts. Brain volumes were scaled to body mass, whereas volumes of brain structures were normalized to individual brain volumes. Normalized brain volume was similar in WWCPS and BN, but lower in WI. Normalized neocortex volume was smaller in both laboratory strains than in WWCPS and the visual cortex was smaller in albino WI rats than in WWCPS and BN. Relative volumes of phylogenetically older structures, such as hippocampus, amygdala, nucleus accumbens and olfactory nuclei, also displayed certain strain-related differences. The present data shows that selective breeding of laboratory rats markedly affected brain morphology, the neocortex being most significantly altered. In particular, albino rats display reduced volume of the visual cortex, possibly related to retinal degeneration and the development of blindness.

## Introduction

Several strains of laboratory rats are bred and supplied for use as experimental animals in multiple research fields including medicine, biology and psychology. These strains are the result of a certain type of domestication of the wild rat species, *Rattus norvegicus*, for which Lockhard [1] proposed 50 years ago the term “laboratorization”. Selective breeding for laboratory purposes has led to remarkable differences between laboratory rats and their wild ancestors in the morphology, physiology and behavior. As recognized by Lockhard [1], laboratorization has resulted in the elimination of numerous traits maladaptive in a laboratory environment, such as savageness and the fight and flight response, and active selection of genetic background of desired traits such as amiability, acceptance of strangers, larger litters, long reproductive periods, tolerance to loud noises, etc. Further differences between laboratory and wild rats, which have been characterized more recently, include differences in complex behaviors such as burrowing, swimming [2] and play-fighting [3, 4]. Laboratory rats learn quicker than wild rats but they also are faster in forgetting the learned reaction [5, 6]. Wild rats exhibit a significantly higher level of aggression towards other individuals of their species [7, 8], and are more responsive to changes in their environment [2, 9–11].

The captured albino mutant individuals of the wild *Rattus norvegicus* were one of the earliest type of rats domesticated for scientific purpose. One of the reason could have been related to the pleiotropic association of melanin-based lighter coloration to certain attributes of social behavior such as tameness [12, 13]. The albino rats have become extremely popular in experimental psychology, neurology and physiology in the mid-20^th^ century [14]. Studies on albino rats have provided substantial part of knowledge about the functioning of the nervous system. Currently the majority of rats bred for laboratory purposes are albino.

There is a significant variability in visual acuity among laboratory rat strains. Prusky [15] observed that in laboratory pigmented rats domestication does not seem to have negatively affected visual acuity, which may even have been enhanced by selective breeding. However, albinism by itself leads to a severe and sustained impairment of visual acuity, associated with a lack of light-protective pigmentation inside the eyeballs, which gradually leads to the degeneration of the retina, linked to changes in the visual cortex connectivity [16].

The aim of this study was to compare volumes of selected brain structures in wild rats and their laboratorized counterparts, albino and pigmented, in order to relate structural differences to the differences in behavior. For this purpose, high resolution *in vivo* magnetic resonance imaging (MRI) of brain structure in the wild-captive and laboratory rats was performed. Non-invasive brain MRI provides an excellent method for studies of rodent brain structure. Compared with traditional histology, *in vivo* MRI studies make it possible to avoid artifacts generated by procedure of removing brain from the scull and by multistep histological processing. Moreover, MRI allows to employ digital atlases that have numerous advantages over their traditional paper counterparts [17], in particular may be used for automatic segmentation of images using a standardized ("stereotaxic") three-dimensional coordinate frame [18]. The use of *in vivo* MRI techniques for comparative studies may allow to substantially reduce the number of animals required to obtain statistically meaningful data sets, being compliant with the 3R principle formulated by Russell and Burch [19].

## Materials and methods

### Animals

All animal procedures were approved by the 4^th^ Local Ethics Committee for Animal Experimentation in Warsaw (Permission Number: 39/2014). The procedures were carried out in strict accordance with the recommendations in the Guide for the Care and Use of Laboratory Animals of the National Institutes of Health and documented in compliance with the ARRIVE guidelines. All efforts were made to minimize suffering.

All animals used in the study were maintained in standard conditions in accordance with the requirements of the Directive 2010/63/EU of the European Parliament and of the Council on the protection of animals used for scientific purposes.

For imaging studies young adult male rats at the same age (8–9 week old) were sourced from the following three populations:

1. The Warsaw Wild Captive Pisula-Stryjek rats (WWCPS, n = 9), considered as a model of population for the wild *Rattus norvegicus*, were obtained from a colony maintained in the facility of the Institute of Psychology, Polish Academy of Sciences, Warsaw, Poland. The WWCPS rats were derived in 2006 from sample originating from five independent colonies of feral rats [20]. In order to prevent the development of domestication in the breeding colony, new genetic material from rats captured freshly in a variety of locations has been systematically introduced to the colony. To avoid the stress response to experiment only the second and third generation (F2-F3) of laboratory-reared WWCPS wild rats were selected.
2. Brown Norway BN/CrlCmd (BN, n = 8) originated from the inbred strain maintained at the Mossakowski Medical Research Centre, Polish Academy of Sciences, Warsaw, Poland (MMRC). The parent pairs were obtained in 1997 from the Charles River Laboratories, Germany.
3. Wistar Cmd:WI(WU) (WI, n = 8) originated from outbred stock maintained at MMRC. The parent pairs were obtained in 1987 from Charles River Laboratories, Germany.

### Magnetic resonance images acquisition and processing

MR brain imaging was performed with a Bruker BioSpec 70/30 Avance III 7T system, equipped with a transmit cylindrical radiofrequency coil (8.6 cm inner diameter) and a rat brain dedicated receive-only array coil (2x2 elements) positioned over the animal’s head. The animals were anaesthetized with isoflurane (4% for induction, 1.5–2% in oxygen for maintenance, supplied through a mask), and placed on a dedicated rat scan bed in the prone position with the head fixed in a stereotactic apparatus with plastic retainers for the teeth and ears (Bruker, Germany). Physiological monitoring, including respiration rate and body temperature, was performed throughout the imaging sessions.

Structural transverse MR images covering the whole brain were acquired with the T2-weighted TurboRARE sequence (TR/TEeff = 5000/30ms, RARE factor = 4, spatial resolution = 125μm x 125μm x 500μm, 54 slices, no gaps, number of averages = 3). The structural images were exported in DICOM format (.dcm) and then converted to NifTI format (.nii).

For the segmentation of individual brain image structures we employed the *in vivo* rat atlas (created from images of the brains of 97 male Sprague-Dawley rats) and published by Schwarz et al. [21] that provides template image covering the whole brain and labelled images of 50 cortical and subcortical delineated brain structures based on the rat brain atlas by Paxinos and Watson [22]. To improve accuracy of structure delineation we transformed the Schwarz template image and the labelled image into tissue probability maps space associated with the template image provided by Valdes-Hernandez et al. [23] that is based on *in vivo* images of the rat brain.

**Fig 1:**
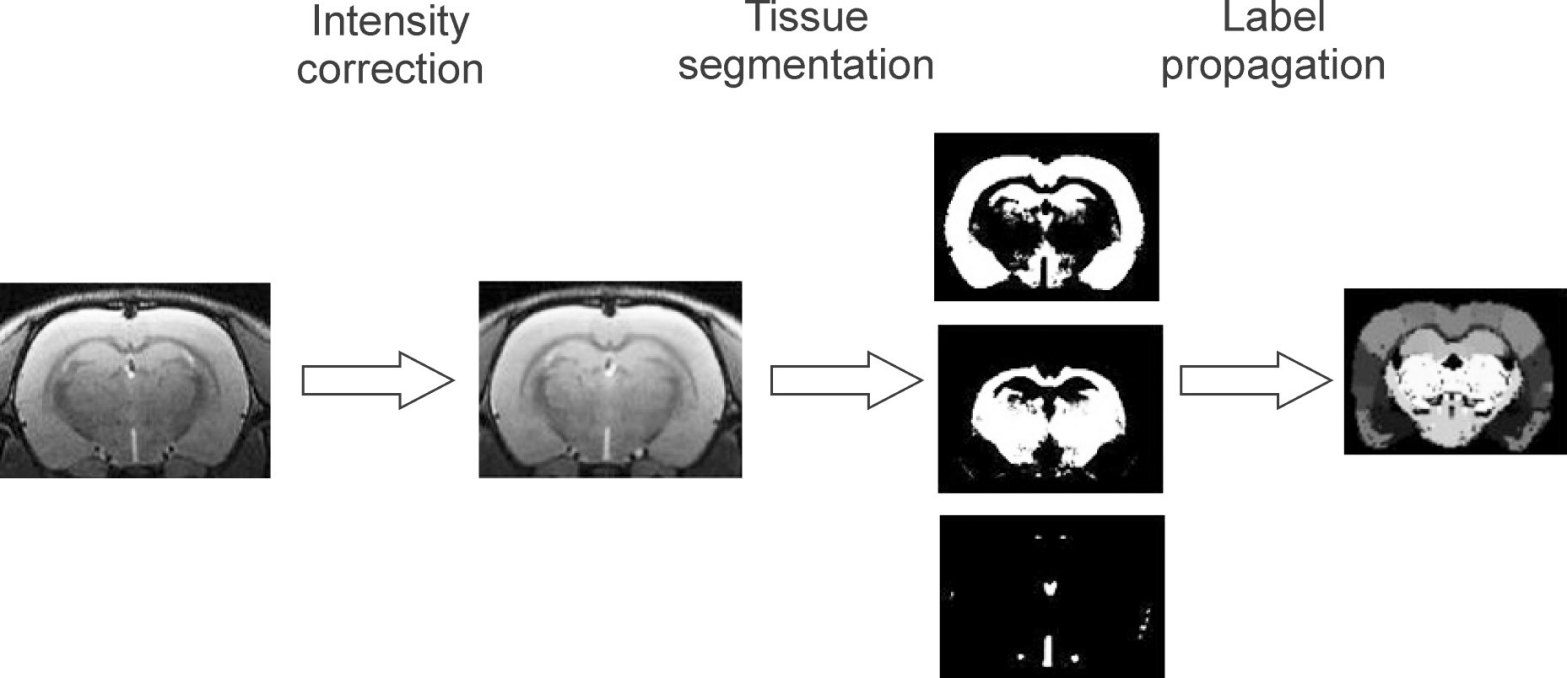
A simplified image processing pipeline. High resolution T2-weighted images were intensity corrected and automatically segmented for white matter, grey matter and cerebrospinal fluid. In next step the images were transformed to brain template which allowed applying labels (ie. identification of brain structures defined in brain atlas). Back-transformation to original dimension allowed identification of brain structures in the original images and calculation of volumes of the pre-defined structures.

The analysis of the image data was performed in the following steps (Fig 1):

1. ***Intensity correction*.** An intensity bias field correction was performed on the data using N4ITKBiasFieldCorrection, a tool distributed with the 3D Slicer software [24].
2. ***Image registration*.** After intensity, non-uniformity correction was implemented with the same origin of the coordinate system as in the template image was set for each individual image. Using tissue priors, each subject image was segmented using SPM8 into grey matter, white matter and cerebrospinal fluid. This method additionally provides forward and inverse transformation to the template space.
3. ***Structures labelling***. The inverse transformations obtained from the image registration (step 2) were then used to warp labelled atlas images into the space of each individual subject image using the IBASPM toolbox for SPM5 [25].
4. ***Volumetric measurements*.** Absolute volumes for subject segmented structures and the whole brain volume were calculated by multiplying number of the voxels belonging to the structure by voxel volume [26, 27]. Scaled (normalized) volumes were obtained by dividing structure volume by whole brain volume.

The segmentation accuracy was validated by comparing the results of automated segmentation with the results of manual segmentation of the hippocampus. Manual segmentation was performed in 3D Slicer software (version 4.8.1, www.slicer.org). The volumes of automatically segmented hippocampi were compared with those of manually segmented hippocampi according to Badea et al. [28]. Volume difference (VD) was calculated as follows:

$$VD=\frac{2*|V_{manual}-V_{auto}|}{V_{manual}+V_{auto}}*100$$

The olfactory bulb (and the accessory olfactory bulb) was manually segmented in 3D Slicer according to the Paxinos atlas [29] and Paxinos MRI/DTI atlas [30].

### Statistical analysis

We used STATISTICA for Windows (version 10, StatSoft, Inc., Tulsa, OK, USA, www.statsoft.com). Levene’s test revealed significant differences in variances for scaled brain volume and several brain structures (retrosplenial cortex, accumbens shell, bed nucleus stria terminalis, corpus callosum, mesencephalic region, raphe; S3 Table), therefore non-parametric tests were chosen. Significances of differences between groups were evaluated with the Kruskal-Wallis test followed by U Mann-Whitney test. Critical levels of significance were corrected for multiple comparisons with Bonferroni method. For overall significance level α = 0.05 and testing three (m = 3) hypothesis, then we tested each individual hypothesis at α = 0.05/3 = 0.01(6) according to the equation:

$$p_{i}<\frac{\alpha }{m}$$

## Results

**Fig 2:**
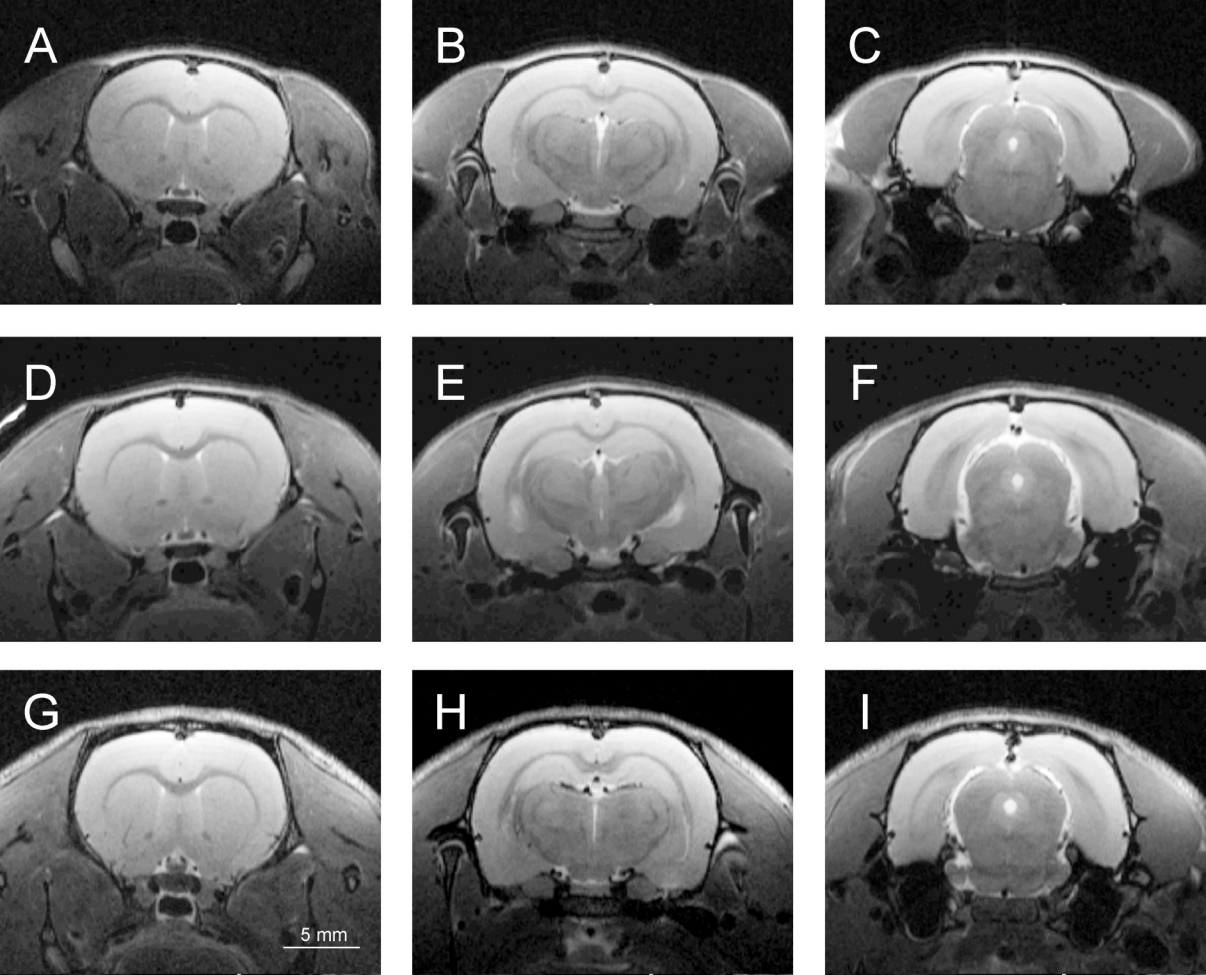
Representative coronal T2-weighted MR images of WWCPS (A-C), BN (D-F) and Wistar rat (G-I).

**Fig 3:**
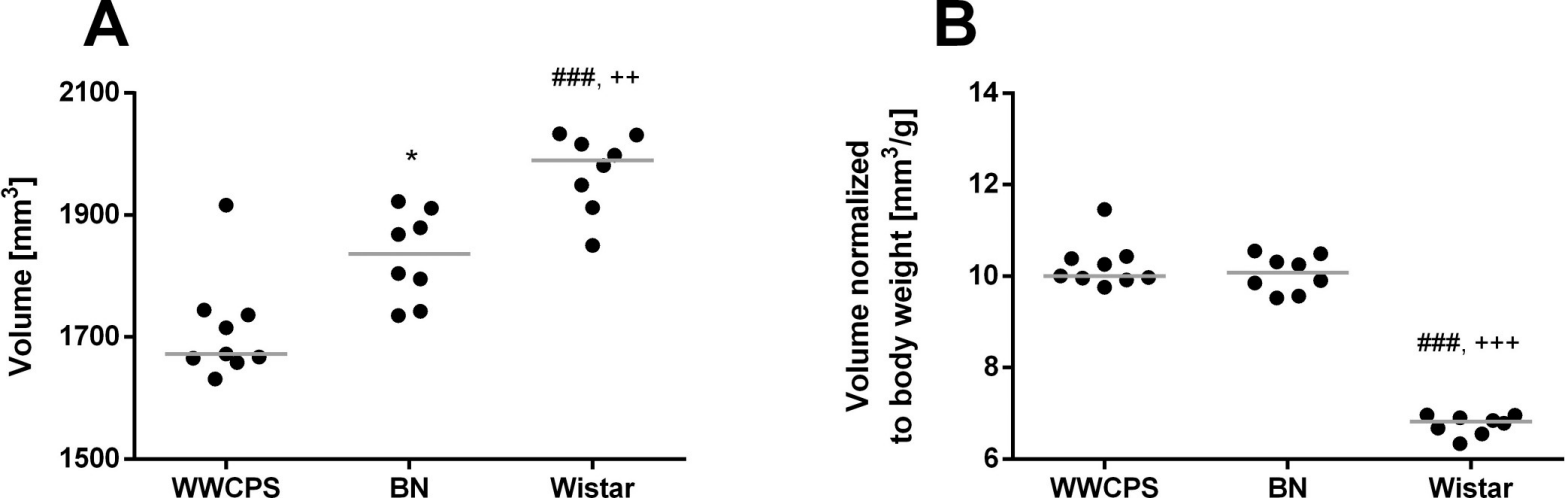
**Brain size in WWCPS, BN and Wistar rats.** (A) Absolute (ie. non-normalized) volumes expressed in mm^3^. (B) Ratio of brain volume to body weight (normalized values) in WWCPS, BN and Wistar rats. Lines represent medians, dots represent individual values Symbols represent Bonferroni corrected significance levels: *p<0.017 BN vs. WWCPS; ^###^p<0.0003 Wistar vs. WWCPS; ^++^p<0.003, ^+++^p<0.0003 BN vs. Wistar.

The tested groups of rats presented large differences in body weight: WWCPS (167.3±9.1 g), BN (182.5±7.2 g). Wistar rats had significantly higher body mass than WWCPS (292.0±10.2 g; p = 0.00003) and BN (p = 0.00008). Structural T2-weighted MR images were acquired for all the tested groups of animals (Fig 2) and were used for calculations of the brain size and volumes of the specific brain structures. Mean brain size in WWCPS was calculated to 1712±28.5 mm^3^. It was smaller than in BN (1832± 25.9 mm^3^; p = 0.01) and WI (1971±22.7 mm^3^, p = 0.0003). Brain volume scaled to body mass was similar in WWCPS and BN (10.24±0.17 mm^3^/g vs. 10.05±0.14 mm^3^/g; ns) but significantly lower in WI (6.75±0.22 mm^3^/g; p = 0.00008; Fig 3, Table 1).

**Table 1:** Mean brain to body weight ratio (±SD) and mean percentage of various brain structures in WWCPS, BN and Wistar rats (ie. normalized data).

Structures	WWCPS	BN	Wistar
***Brain (scaled to body weight)***	10.24±0.51	10.05±0.40 (-1.77%)	6.75±0.22 (-34.05%) ^###^,^+++^
***Total cortex***	44.47±0.36	43.85±0.51 (-1.39%) *	43.71± 0.29 (-1.71%) ^##^
Auditory Cortex	3.00 ±0.34	2.94±0.03 (-2.04%) *	2.96±0.03 (-1.13%)
Cingulate Cortex	2.53±0.03	2.54±0.04 (0.18%)	2.53±0.04 (-0.06%)
Entorhinal Cortex	2.87±0.03	2.94±0.04 (2.33%) *	3.00±0.03 (4.42%) ^###^, ^+^
Frontal Cortex Association	2.07±0.02	2.05±0.02 (-1.10%)	2.07±0.01 (-0.06%)
Insular Cortex	2.79±0.03	2.76±0.04 (-0.96%)	2.78±0.03 (-0.23%)
Medial Prefrontal Cortex	2.27±0.02	2.26±0.02 (-0.09%)	2.26±0.02 (-0.42%)
Motor Cortex	5.67±0.07	5.53±0.11 (-2.62%) **	5.51±0.10 (-2.83%) ^##^
Orbitofrontal Cortex	2.29±0.02	2.28±0.04 (-0.39%)	2.30±0.03 (0.53%)
Parietal Cortex Association	1.96±0.02	1.92±0.02 (-2.01%) **	1.91±0.01 (-2.22%) ^##^
Piriform Cortex	2.55±0.03	2.52±0.03 (-0.94%)	2.54±0.03 (-0.06%)
Retrosplenial Cortex	2.71±0.06	2.70±0.07 (-0.55%)	2.60±0.03 (-4.05%) ^##^,^+^
Somatosensory Cortex	9.10±0.14	8.94±0.16 (-1.82%) *	8.93±0.19 (-1.87%)
Temporal Cortex Association	1.14±0.03	1.14±0.02 (-0.73%)	1.11±0.01 (-3.02%) ^##^,^++^
Visual Cortex	3.51±0.17	3.34±0.08 (-4.79%)	3.19±0.03 (-9.27%) ^###^,^+++^
***Hippocampus***	6.54±0.08	6.74±0.07 (3.08%) ***	6.77±0.09 (3.44%) ^###^
Antero Dorsal	1.30±0.04	1.32±0.03 (1.66%)	1.35±0.02 (3.98%) ^##^
Posterior	0.752±0.010	0.776±0.018 (3.23%) **	0.771±0.013 (2.64%) ^#^
Postero Dorsal	1.92±0.03	1.93±0.03 (0.86%)	1.91±0.03 (-0.08%)
Subiculum	1.14±0.03	1.19±0.03 (4.57%) **	1.18±0.03 (3.43%)
Ventral	1.44±0.03	1.53±0.02 (6.06%) ***	1.56±0.02 (8.05%) ^###^,^+^
***Thalamus***			
Dorsolateral	1.70±0.03	1.68±0.02 (-1.33%)	1.69±0.03 (-0.38%)
Midline Dorsal	0.761±0.021	0.738±0.016 (-2.98%)	0.777±0.026 (2.09%) ^+^
Ventromedial	0.274±0.007	0.268±0.005 (-2.20%)	0.272±0.008 (-0.74%)
***Hypothalamus***			
Lateral	0.832±0.015	0.865±0.027 (4.05%) **	0.851±0.015 (2.33%)
Medial	1.33±0.03	1.41±0.08 (5.72%) *	1.38±0.03 (3.46%)
***Nucleus Accumbens***			
Core	2.55±0.3	2.57±0.02 (0.63%)	2.59±0.01 (1.40%) ^#^
Shell	2.13±0.02	2.16±0.02 (1.15%)	2.17±0.01 (1.76%) ^###^
***Other structures***			
Amygdala	3.13±0.05	3.24±0.04 (3.43%) **	3.23±0.05 (3.12%) ^##^
Bed Nucleus Stria Terminalis	1.83±0.02	1.84±0.01 (0.15%)	1.85±0.01 (1.03%) ^#^
Caudate Putamen	5.74±0.13	5.87±010 (2.38%)	5.89±0.11 (2.65%)
Corpus Collosum	3.18±0.02	3.21±0.04 (0.70%)	3.21±0.07 (0.74%)
Diagonal Band	0.636±0.006	0.652±0.015 (2.53%) *	0.648±0.007 (1.80%) ^#^
Globus Pallidus	0.751±0.009	0.768±0,011 (2.36%) *	0.759±0.013 (1.13%)
Internal Capsule	0.797±0.013	0.810±0.012 (1.75%)	0.804±0.01 (0.94%)
IPAC	0.496±0.007	0.505±0.011 (1.71%)	0.503±0.009 (1.43%)
Medial Geniculate	0.537±0.007	0.548±0.011 (1.97%)	0.550±0.010 (2.44%) ^#^
Mesencephalic Region	1.28±0.02	1.28±0.04 (-0.28%)	1.32±0.04 (3.18%)
Olfactory Nuclei	0.691±0.010	0.702±0.012 (1.64%)	0.717± 0.010(3.70%) ^##^
Olfactory Tubercle	0.593±0.009	0.600±0.011 (1.27%)	0.608±0.010 (2.64%) ^##^
Periaqueductal Grey	0.679±0.016	0.667±0.019–1.78%)	0.672±0.016 (-1.07%)
Pons	2.43±0.09	2.52±0.29 (3.94%)	2.46±0.09 (1.34%)
Raphe	0.468±0.009	0.465±0.011 (-0.71%)	0.471±0.006 (0.51%)
Septum	0.768±0.028	0.776±0.020 (1.06%)	0.794±0.034 (3.32%)
Substantia Innominata	0.446±0.005	0.443±0.007 (-0.75%)	0.445±0.006 (-0.43%)
Substantia Nigra	0.552±0.010	0.560±0.11 (1.32%)	0.562±0.010 (1.82%)
Superior Colliculus	1.33±0.02	1.34±0.03 (0.81%)	1.31±0.02 (-1.79%)
Ventral Pallidum	0.242±0.004	0.240±0.007 (-0.72%)	0.238±0.004 (-1.60%)
Ventral Tegmental Area	0.232±0.004	0.229±0.004 (-1.22%)	0.228±0.002 (-1.33%)
Zona Incerta	0.261±0.005	0.259±0.007 (-0.70%)	0.258±0.002 (-1.25%)
Medulla	3.23±0.08	3.305±0.116 (2.28%)	3.32±0.07 (2.72%)
Cerebellum	9.11±0.25	8.90±0.22 (-2.23%)	8.96±0.14 (-1.63%)

In brackets difference vs. WWCPS expressed in percentages. Symbols represent Bonferroni corrected significance levels

*p<0.017

**p<0.003

***p<0.0003 WWCPS vs. BN

^#^p<0.017

^##^p<0.003

^###^p<0.0003

^####^p<0.00003 WWCPS vs. Wistar

^+^p<0.017

^++^p<0.003

^+++^p<0.0003 BN vs. Wistar.

Significant differences vs. WWCPS are highlighted in shades of red (values greater than in WWCPS) or shades of blue (values smaller than in WWCPS), intensity indicates significance levels.

**Fig 4:**
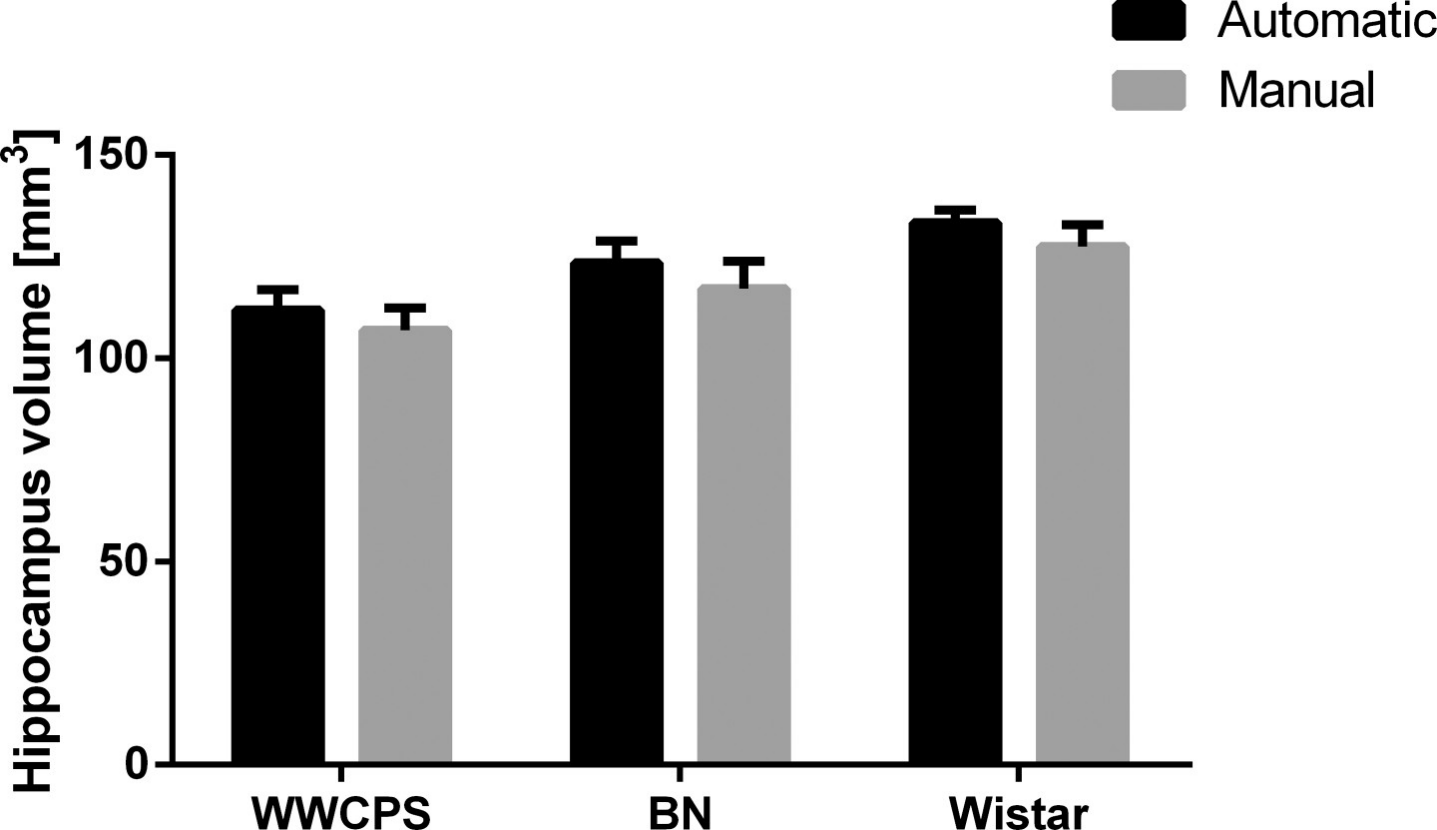
Segmentation validation. Results of automated segmentation (black bars) was compared with manual segmentation of the hippocampi (grey bars) for all the individuals included in the study. The graph presents non-normalized data. Means ± SD.

Accuracy of automatic segmentation was validated by comparison of the results of automated segmentation of the hippocampus with the results of manual segmentation of this structure (Fig 4, S2 Table, S1 Fig). The percentage volume differences (VD) between the manual and automated segmentation were 4.82±3.22% (mean±SD) for WWCPS, 6.04±4.33% for BN and 4.71±3.86% for Wistar. There were no significant differences in VD between the groups (p = 0.52, Kruskal-Wallis test).

Mean brain volumes and volumes of virtually all the analyzed brain structures were bigger in Wistar rats than in WWCPS (S1 Table). The one remarkable exception (no significant difference between Wistar and WWCPS) was the visual cortex. Several brain structures were bigger in BN than in WWCPS, eg. hypothalamus.

**Fig 5:**
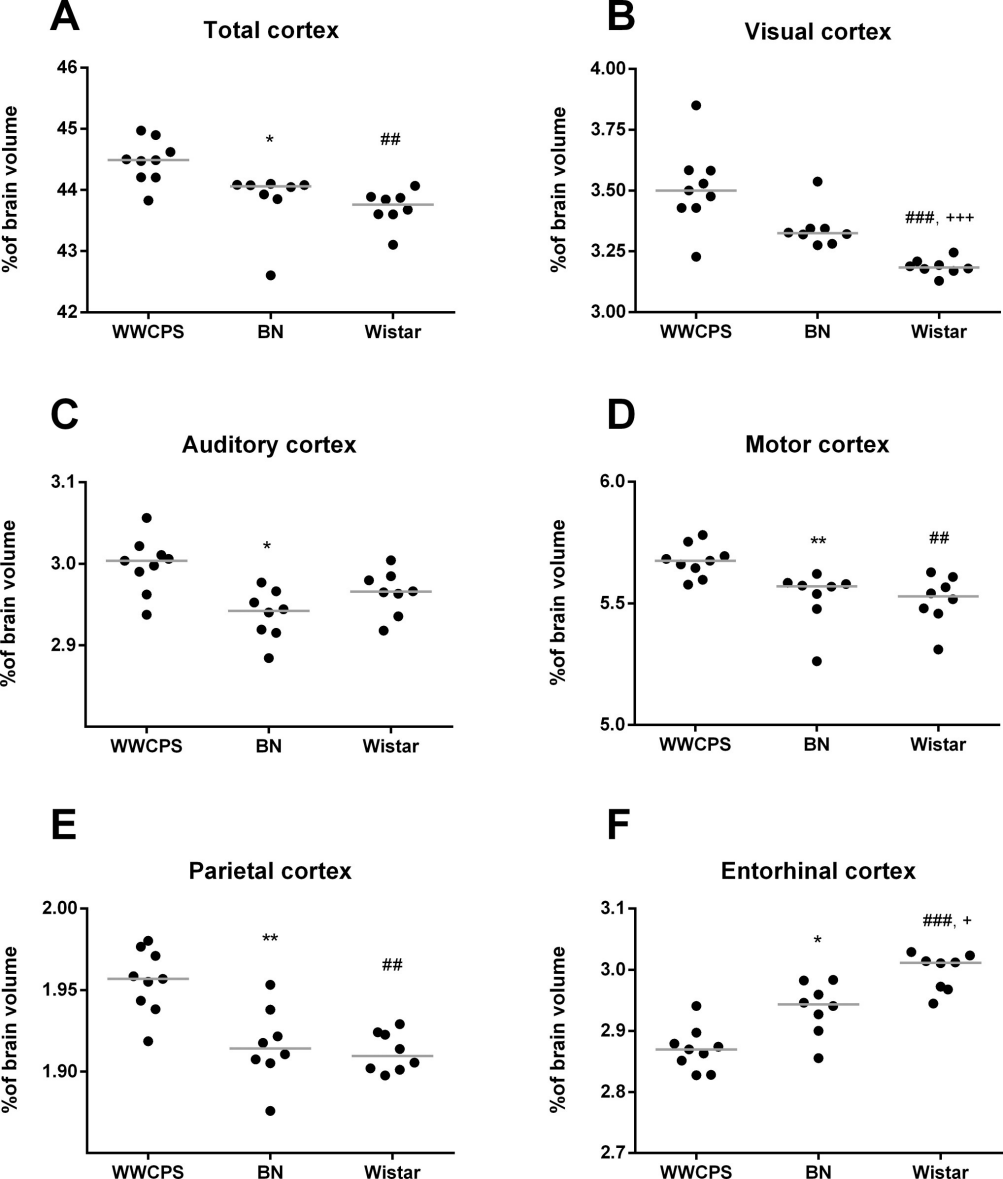
Relative volumes (normalized to individual brain volumes) of cortical brain structures in WWCPS, BN and Wistar rats. Lines represent medians, dots represent individual values. Symbols represent Bonferroni corrected significance levels: *p<0.017, **p<0.003 BN vs. WWCPS; ^##^p<0.003, ^###^p<0.0003 Wistar vs. WWCPS; ^+^p<0.017, ^+++^p<0.0003 BN vs. Wistar.

Normalized volumes of the brain structures (expressed as percentage of the brain volume) allowed us to compare differences in the brain proportions. The average fraction of the total neocortex (expressed as percentage of individual brain volume) was bigger in WWCPS (44.47±0.12%) than in WI rats (43.71±0.10%; p = 0.001) and in BN (43.85±0.51%; p = 0.004; Fig 5, Table 1). There were also a reduction in numerous cortical structures volume in albino rats and fewer in BN rats as compared to WWCPS (Fig 5, Table 1). Only one cortical structure, entorhinal cortex was over 4% bigger in Wistar rats than in WWCPS (p = 0.00008). The most prominent difference was noted for visual cortex that was over 9% smaller in albinotic Wistar rats than in WWCPS (3.19±0.01% vs. 3.51±0.06%, p = 0.0002) and 4% smaller than in Brown Norway (WI 3.19±0.01% vs. BN 3.34±0.03%; p = 0.0002) but no difference was observed between pigmented rats: BN and WWCPS (p = 0.13). Less pronounced but significant differences were noted for auditory cortex (BN vs. WWCPS p = 0.004), motor cortex (WWCPS vs. BN p = 0.001; WI vs. WWCPS p = 0.001), parietal cortex (BN vs. WWCPS p = 0.002; WI vs. WWCPS p = 0.0006), retrosplenial cortex (WI vs. WWCPS p = 0.0006; BN vs. WI p = 0.007) and temporal cortex (WI vs. WWCPS p = 0.002; BN vs. WI p = 0.003).

**Fig 6:**
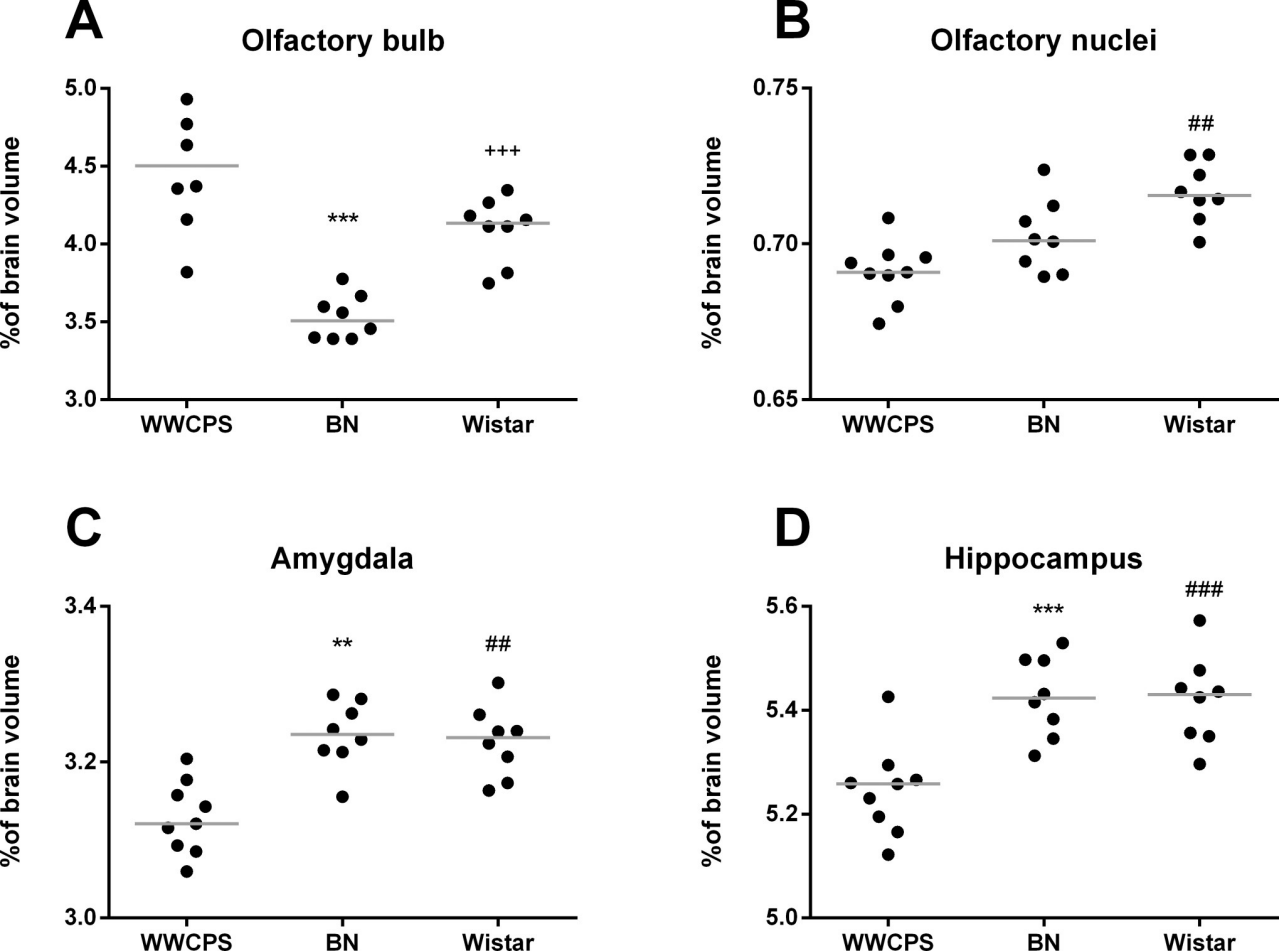
Relative volumes of brain structures (normalized to individual brain volumes) in WWCPS, BN and Wistar rats. Lines represent medians, dots represent individual values. (A) The olfactory bulbs, (B) olfactory nuclei, (C) amygdala and (D) hippocampus. The olfactory bulbs were segmented manually and the other structures were segmented automatically. Symbols represent Bonferroni corrected significance levels: ** p<0.003, ***p<0.0003 BN vs. WWCPS; ^##^p<0.003, ^###^p<0.0003 Wistar vs. WWCPS; ^+++^p<0.0.0003 BN vs. Wistar.

On the other hand, numerous brain structures besides cerebral cortex were relatively bigger in laboratory strains than in WWCPS rats (Fig 6, Table 1). Relative hippocampus volume was greater in both laboratory strain than in wild rats (WI vs. WWCPS p = 0.0003 and BN vs. WWCPS p = 0.0003), in particular the ventral hippocampus that was over 8% bigger in WI than in WWCPS (p = 0.00008). The relative volume of amygdala was also significantly bigger in both laboratory strains when compared to wild rats: BN (3.24±0.02%, p = 0.0006) and WI (3.23±0.02%; p = 0.001) than WWCPS (3.13±0.02%). Relative volume of olfactory tubercle and olfactory nuclei was significantly higher in WI than in WWCPS (p = 0.006 and p = 0.0003, respectively). The relative volumes of the olfactory bulbs were smaller in BN than in WWCPS (WWCPS 4.55±0.49%; BN 3.53±0.14%, WWCPS vs. BN p = 0.0003) and in Wistar (WI 4.09±0.21%; BN vs. WI p = 0.0003). There was no significant difference between WWCPS and Wistar.

## Discussion

In the present study we used *in vivo* MRI technique employing a magnetic field of 7 Tesla to collect images of brains of young adult wild-captive rats and young adult rats of two laboratory strains, albino (Wistar) and pigmented (Brown Norway). Animals were randomly selected from different families by age (8–9 weeks). In the images, using digital MRI Schwarz rat brain atlas, we segmented and quantitated 50 brain regions, and looked for differences between the strains. Fully automated MR image analysis and quantitation of structure volumes provided fast, all-encompassing and non-invasive quantitative evaluation of brain structures. In the case of rat brain poor contrast between anatomical structures makes it difficult to achieve reproducible results using manual segmentation, whereas the standardized image registration and warping methods make the segmentation process more precise and objective [25–27]. Atlas-based morphometry approach allowed to avoid operator bias, providing for faster and more accurate assessment of brain morphology. Moreover, MR imaging performed *in vivo* allowed assessment of the size of brain structures in their natural environment and conditions, thus avoiding post-mortem changes in shape and volume caused by the preparation or fixation of the tissue [26, 31]. The brain images were registered to the templates and atlases basing on images of a similar spatial resolution that were acquired *in vivo* [21, 23].

Automated MR atlases are relatively widely used for high throughput phenotyping of various strains of laboratory animals [32, 33]. Both traditional and electronic atlases are used for animals of different strains on the condition that they represent the same maturation state and do not display severe anatomical abnormalities [21, 29]. In particular, the Paxinos atlas [29] was created for the Wistar rat but is widely used also for other rat strains. The Schwarz atlas that we use in this study was already evaluated for a large group of male Sprague-Dawley rats (n = 97) and the identification of the structures was based on the Paxinos atlas. Schwarz et al. analyzed the template registration quality on brain images of separate group of 16 animals and provided a detailed analysis of the success of registration of these images to the template. Numerous studies demonstrate successful registration of images obtained from different rat strains to this template [34, 35]. Nevertheless, we performed validation of our results that based on comparison of manual vs. automatic segmentation of hippocampus [28]. This analysis showed small differences between the results of manual and automatic segmentation. Moreover, the accuracy of segmentation did not seem to depend on the group of the animals tested. It cannot be excluded some of the structures are segmented with poorer accuracy, in particular the smaller structures [26].

### Domestication-related changes in brain size

The rats from all populations were in the same maturation state, ie. young adults. However, the weights of the animals were significantly different as expected. The differences in weight between different rat strains are well known and our weight records are in good agreement with those provided by professional animal suppliers [36, 37].

Brain volume was bigger in rats with greater body weight, ie. Wistar>BN>WWCPS. However, brain to body weight ratio was similar in BN and WWCPS rats but smaller in Wistar. Brain size usually increases with body size, but most domesticated animals often have a greater body weight and relatively smaller brain volume than their wild counterparts [38–41]. Some of the structural brain differences may be associated with inbreeding and albinism. While, Wistar are the one of the oldest albinotic laboratory outbred stock, BN is an inbred strain that received a significant genetic contribution from wild rat [42]. The laboratory strains also differ among themselves in behavior. The small differences in brain volume between WWCPS and BN rats may result from the much shorter time of BN domestication than WI rats.

As brain size in animals generally increases with body weight, it could be assumed that body weight and brain size are tightly related. For this reason evolutionary and allometric studies in animals frequently base on relative brain size rather than absolute brain size [41, 43]. Because of the difference in the total brain size, comparison of “absolute” volumes of the segmented brain structures was not very informative: virtually all brain structures were bigger in Wistar rats than in WWCPS. To detect possible differences in the brain proportions within the animal groups, we have presented volumes of the segmented brain structures as a percentage of total brain volume.

### Domestication-related changes in regional brain morphology

Comparing brain structures in wild-captive rats with laboratory rats we detected, a reduction in the brain volume to body weight ratio in WI and noticed that the changes in proportions of particular brain structures do not appear homogeneously but rather in a mosaic fashion. Some cortical structures seem to be more affected by domestication than other areas developed at earlier phylogenetic stages. We analyzed 50 segmented brain regions based on the MRI Schwarz atlas and extensively studied the structures which could be coupled with biobehavioral profile changes during the domestication process such as lower aggression, socio-positive maternal behavior and cognitive abilities, as well as less hormonal stress responses [44]. These changes are caused by systematic selection made by humans and adaptations to the exposure to strictly defined artificial environments with standardized diet, low level of external stimuli or without threat from the predators.

The relative total cortex volume in laboratory rats was smaller than in wild rats. After cortex segmentation there were a reduction in almost all of cortical structures volume in albino Wistar rats and in few in BN. In particular we observed significant differences in some functionally related parts of cortex, especially in the area responsible for processing sensory information (visual, retrosplenial, parietal and auditory cortex) or controlling movements (motor cortex). These areas are essential for the survival of animals in the wild and were significantly decreased in laboratory rats. Similarly, decrease in total cortex and areas involved in processing of the sensory information and motor system was found both in early domesticated mammals (eg. guinea pig *Cavia apera f*. *porcellus*, pig *Sus scrofa*, llama *Lama glama*, sheep *Ovis ammon*) and in the relatively recently domesticated species (eg. gerbil *Meriones unguiculatus* or mink *Mustela vison energumenos*) [40, 44]. Only the entorhinal cortex which is considered to be periallocortex and is involved in spatial learning in rodents was significantly larger in WI rats compared to wild rats.

### Changes in structures related to sensory processing and motor functions

Many laboratory strains of this species are albinotic and develop various ocular pathologies resulting in impaired visual acuity [45] as well as structural abnormalities in the connections of the occipital cortex [16]. Importantly, abnormalities in the functioning of the visual system are manifested already in very young animals (eg. 1.5 months old Wistar rats) [46]. We observed the largest reduction in the relative volume of the visual cortex in albino Wistar rat when compared with both pigmented rats (WI and WWCPS about 9% and 4% between WI and BN) whereas, there was no difference between WWCPS and BN. Also, the retrosplenial and temporal association cortex which border and have functional connections with the visual cortex presented a smaller volume in WI then in pigmented rats. Our study confirms that changes in visual acuity in albinotic Wistar rats lead to structural changes in the vision-related areas. This diminution of visual structures could be a remarkable example for conspecific variability and brain plasticity. This fact should be considered during selecting animal models for neurological studies, especially for behavioral tests associated with vision.

We detected only slight differences in somatosensory cortex (that is involved in processing of proprioception and touch, including whiskers sensory system) volume between BN and WWCPS. However the sensory association areas, such as, the parietal cortex that integrates sensorimotor information among various modalities (visual, auditory, whiskers) was significantly decreased in laboratory rats. The relative volume of the of auditory area in cerebral cortex was also significantly smaller in the BN than in WWCPS. Such a notable reduction was not observed in albino WI rats. The principal auditory relay, the medial geniculate nucleus was even slightly enhanced in WI.

In rats, smell plays an important role in recognition of their offspring and partners or predators and searching for food. In domesticated animals, the components of the olfactory system (in particular the olfactory bulbs) are usually reduced. [43, 47].

In our study the olfactory bulbs (OB) were significantly smaller in laboratory pigmented BN strain than in WWCPS rats but not in the albino Wistar rat. Other structures involved in the sense of smell such as olfactory nuclei and olfactory tubercles and entorhinal cortex were relatively bigger in WI than WWCPS but not in the BN rat.

The larger olfactory- and auditory-related brain structures in albino rats than in pigmented animals could be a result of compensation for the visual impairment, i.e. activation the mechanisms of cross-modal neuroplasticity.

In the natural environment wild animals have to escape from predators, explore large areas to find food, shelter or mates. Laboratory rats show less exploratory behavior than their wild conspecifics [48]. The motor cortex which is the area of the cerebral cortex involved in the planning, commanding and controlling voluntary movements were significantly smaller in laboratory rats when compared to wild rats. Laboratory rats had also noticeably smaller parietal and retrosplenial cortex, structures which has connection with motor cortex and play a role in navigational motor planning. For example, it was shown that destruction of a part of the retrosplenial cortex resulted in worse spatial movement parameters such as running speed in rats [49]. The reduction of motor areas in the cortex was also noted in other domesticated mammals [50]. It could be explained by breeding in restricted space with limited ability to perform motor activities.

### Changes in structures possibly related to emotions and stress reaction

Artificial selection during domestication leads to consolidation of traits that are more advantageous and desirable for humans. Namely, animals are selected which present less aggression toward people and other animals, more sociable behavior, high fecundity, and diminished stress responsiveness caused by crowding in cages and restriction of movement. Our study shows significant differences in the relative volume of the entorhinal cortex, amygdala, hippocampus and nucleus accumbens in laboratory rats compared to their wild counterparts. These structures are involved in the regulation of various functions including memory and emotional response such as aggression, fear or motivation. The most significant differences in these structures were observed between WWCPS and Wistar rats. Notably, Wistar stock is characterized by extremely docile behavior and high fertility.

Hippocampus was relatively larger in both laboratory strains than in wild rats. Dorsal hippocampus, which in rodents is involved mainly in spatial memory [51], was slightly bigger. However, the most significant differences were found between the WI and WWCPS rats in relative volume of the ventral hippocampus. This structure, responsible for learning and memory function, is also involved in modulation of anxiety and aggression [52]. Similarly, in farm-bred foxes (*Vulpes vulpes*) selected for tameness Huang et al. showed increased neurogenesis within ventral hippocampus which consequently would lead to enlargement of hippocampal size [53].

Generally, the amygdala has definitely increased in size during evolution process [54]. Significantly higher relative volume in amygdala was noted in both laboratory strains. Similarly, slight increase of relative volume was found, by Kruska for the amygdala volume, in domesticated guinea pig when compared to wild cavies [47]. The increased of relative volume was also shown in WI rats in the core and shell of nucleus accumbens. These structures are involved in the cognitive processing of reward, aggression and sexual behavior in rats. The most pronounced differences were observed in the accumbens shell which is strongly connected to amygdala.

### Changes in structures related to memory and learning

Until recently, it was considered that due to domestication the brain size is reduced and animals lose a lot of their cognitive and learning abilities. However, a recent study on spatial learning with use the Morris water maze shows that some domestic animals such as Guinea pig or Mongolian gerbil [44, 55] have equivalent or even better learning performance than their wild ancestors. These findings are similar to those on rats, in which the laboratory rats might also outperform the wild rats in spatial learning and memory tasks [56, 57]. Domesticated animals including laboratory rats seem to adapt faster to man-made environment and better cooperate with the experimenter, whereas wild animals exhibit excessive emotional responses and they are more susceptible to stress during the test. For this reason wild animals can be worse in solving spatial memory tasks in laboratory conditions. On the other hand, they are more effective in complicated tasks that require a higher cortical coordination (wild cavies, gerbils, foxes, dogs) [58, 59].

In our study we find that relative brain volume was smaller in laboratory WI rats. Particularly some cortical regions involve in the cognitive and memory functions including visuospatial orientation and planning the future such as the retrosplenial, temporal associations and parietal association cortex were decreased considerably. Furthermore, phylogenetically older structures such the hippocampus (archeocortex) which play a crucial role in learning and memory processes (especially spatial memory) in rodents, were changed but not as much as the phylogenetically younger cortex structure. Only the enthorhinal cortex (periallocortex) which cooperate with hippocampus by registering information on grid maps of the surrounding area had extraordinarily bigger volume in WI rats. Relative total volume of the hippocampus was significantly bigger in both laboratory strains compared to WWCPS. The differences were observed in the anterodorsal, posterior and ventral hippocampus and subiculum. In particular, the most pronounced difference was observed in the relative volume of the ventral hippocampus between WWCPS and both the laboratory strains.

## Conclusion

Alterations in the brain structure that might be attributed to domestication in rat do not appear homogeneously. Firstly, cortical (isocortex) structures form a definitely smaller part of the brain volume in laboratory rats, especially in Wistar, as compared to WWCPS rats. Secondly, the structures belonging to phylogenetically older layers such as: hippocampus, amygdala, accumbens nuclei, and olfactory tubercle and nuclei form a relatively bigger part of the brain volume in WI and BN rats as compared to their wild-type counterparts. These two robust effects may lead us to propose a hypothesis of the mechanism underlying both of these effects. The prolonged effect of domestication/laboratorization in BN and Wistar rats is manifested in their brain by the relative reduction of cortical volume when compared to the phylogenetically older structures. This would mean, that impoverished, laboratory environmental conditions impair mainly the most recent evolutionary advancements, such as the neocortex and its functions, whereas the more ancient structures remain relatively intact.

## Supporting information

S1 Table Mean brain volumes and mean volumes of the various brain structures in WWCPS, BN and Wistar rats. Non-normalized data.
(DOCX)

S2 Table Validation of the automatic segmentation. (DOCX)

S3 Table Coefficient of variance [%] for different brain structures in WWCPS, BN and Wistar rats. (DOCX)

S4 Table Results of the automatic segmentation of the selected brain structures–non-normalized raw data. (XLSX)

S5 Table Results of the manual segmentation of the olfactory bulb–non-normalized raw data. (XLSX)

S1 Fig Visualization of the manual segmentation of the hippocampus and the olfactory bulbs. (PDF)
