# Supplementary material for: Volumes of brain structures in captive wild-type and laboratory rats: 7T magnetic resonance in vivo automatic atlas-based study
Source: PLoS One. 2019 Apr 11;14(4):e0215348. doi: 10.1371/journal.pone.0215348 (PMC6459519; doi:10.1371/journal.pone.0215348)
Supplement: S1 Table — Non-normalized data. (DOCX) [file pone.0215348.s001.docx]

**S1 Table .** Mean volumes of the brain and various brain structures in WWCPS, BN and Wistar rats. Symbols represent Bonferroni corrected significance levels: *p<0.017 WWCPS vs. BN; ^##^p<0.003, ^###^p<0.0003, ^####^p<0.0003 WWCPS vs. Wistar. Significant differences vs. WWCPS are highlighted in shades of red (values greater than in WWCPS). Intensity indicates significance levels. Mean±SD, non-normalized data.

| **Structures** | **WWCPS** | **BN** | **Wistar** |
| --- | --- | --- | --- |
| ***Brain*** | 1711.6±85.5 | 1832.0±73.2 * | 1971.3±64.3 ^###^,^++^ |
| ***Total cortex*** | 760.93±34.89 | 803.15±29.43 * | 861.61±29.52 ^###^,^++^ |
| Auditory Cortex | 51.32±2.56 | 53.81±2.12 | 58.45±2.22 ^###^,^++^ |
| Cingulate Cortex | 43.34±2.22 | 46.47±1.65 * | 49.90±2.22 ^###^,^+^ |
| Entorhinal Cortex | 49.11±2.40 | 53.78±1.74 ** | 59.06±1.53 ^###^,^+++^ |
| Frontal Cortex Association | 35.50±1.69 | 37.57±1.37 * | 40.86±1.35 ^###^,^++^ |
| Insular Cortex | 47.75±2.39 | 50.61±1.81 * | 54.87±1.82 ^###^,^++^ |
| Medial Prefrontal Cortex | 38.76±1.83 | 41.45±1.50 * | 44.47±1.62 ^###^,^++^ |
| Motor Cortex | 97.10±4.42 | 101.21±4.01 | 108.67±3.47 ^###^,^++^ |
| Orbitofrontal Cortex | 39.19±2.00 | 41.77±1.37 * | 45.37±1.46 ^###^,^++^ |
| Parietal Cortex Association | 33.46±1.47 | 35.10±1.32 | 37.69±1.32 ^###^,^+^ |
| Piriform Cortex | 43.57±1.98 | 46.20±1.50 * | 50.16±1.54 ^###^,^++^ |
| Retrosplenial Cortex | 46.38±2.03 | 49.40±2.41 * | 51.27±1.93 ^##^ |
| Somatosensory Cortex | 155.85±8.86 | 163.7±16.13 | 176.14±7.88 ^##^,^+^ |
| Temporal Cortex Association | 19.58±0.79 | 20.82±1.026 | 21.88±0.70 ^###^ |
| Visual Cortex | 60.03±2.35 | 61.27±3.07 | 62.81±2.08 |
| ***Hippocampus*** | 111.92±4.95 | 123.53±5.31 ** | 133.33±3.11 ^###^,^++^ |
| Antero Dorsal | 22.17±1.32 | 24.11±0.70 * | 26.54±0.89 ^###^,^+++^ |
| Posterior | 12.86±0.62 | 14.22±0.69 ** | 15.20±0.31 ^###^,^+^ |
| Postero Dorsal | 32.77±1.45 | 35.40±1.76 * | 37.72±0. 99 ^###^,^++^ |
| Subiculum | 19.49±0.85 | 21.83±1.14 ** | 23.21±0.56 ^###^,^+^ |
| Ventral | 24.63±1.04 | 27.98±1.24 *** | 30.66±0.76 ^###^,^+++^ |
| ***Thalamus*** | | | |
| Dorsolateral | 29.10±1.55 | 30.73±1.04 * | 33.39±1.10 ^###^,^+++^ |
| Midline Dorsal | 13.02±0.710 | 13.52±0.31 * | 15.31±0.51 ^###^,^+++^ |
| Ventromedial | 4.69±0.27 | 4.92±0.27 | 5.37±0.25 ^##^,^+^ |
| ***Hypothalamus*** | | | |
| Lateral | 14.23±0.74 | 15.86±0.97 ** | 16.78±0.54 ^###^ |
| Medial | 22.85±1.38 | 25.88±2.21 ** | 27.22±1.15 ^###^ |
| ***Accumbens*** | | | |
| Core | 43.64±2.02 | 47.01±1.67 ** | 50.97±1.64 ^###^,^++^ |
| Shell | 36.48±1.68 | 39.50±1.36 ** | 42.76±1.29 ^###^,^++^ |
| ***Other structures*** | | | |
| Amygdala | 53.53±2.40 | 59.27±2.21 ** | 63.59±2.10 ^###^,^+^ |
| Bed Nucleus Stria Terminalis | 31.37±1.57 | 33.63±1.25 * | 36.51±1.25 ^###^,^++^ |
| Caudate Putamen | 98.26±6.32 | 107.57±3.11 * | 116.13±4.97 ^###^,^++^ |
| Corpus Collosum | 54.51±2.75 | 58.78±1.89 * | 63.27±3.27 ^###^,^+^ |
| Diagonal Band | 10.89±0.54 | 11.95±0.64 ** | 12.76±0.38 ^###^ |
| Globus Pallidus | 12.85±0.66 | 14.08±0.58 * | 14.96±0.44 ^###^,^+^ |
| Internal Capsule | 13.63±0.66 | 14.85±0.69 ** | 15.85±0.54 ^###^,^+^ |
| IPAC | 8.49±0.43 | 9.25±0.48 * | 9.92±0.30 ^###^,^+^ |
| Medial Geniculate | 9.19±0.43 | 10.04±0.57 ** | 10.84±0.26 ^###^,^+^ |
| Mesencephalic Region | 21.91±1.11 | 23.39±1.40 * | 26.02±0.80 ^###^,^+^ |
| Olfactory Nuclei | 11.83±0.61 | 12.86±0.44 ** | 14.12±0.36 ^###^,^+++^ |
| Olfactory Tubercle | 10.15±0.53 | 12.00±0.47 * | 11.99±0.34 ^###^,^++^ |
| Periaqueductal Grey | 11.63±0.66 | 12.22±0.54 * | 13.25±0.43 ^##^,^++^ |
| Pons | 41.58±3.25 | 46.31±6.67 | 48.47±1.96 ^##^ |
| Raphe | 8.01±0.47 | 8.52±0.41 * | 9.28±0.31 ^##^,^++^ |
| Septum | 13.15±0.91 | 14.22±0.55* | 15.66±1.06 ^###^,^+^ |
| Substantia Innominata | 7.64±0.35 | 8.12±0.38 * | 8.76±0.26 ^###^,^+^ |
| Substantia Nigra | 9.45±0.46 | 10.26±0.55 * | 11.08±0.32 ^###^,^+^ |
| Superior Colliculus | 22.77±1.15 | 24.56±0.71 | 25.75±0.79 ^###^,^+^ |
| Ventral Pallidum | 4.14±0.26 | 4.40±0.28 | 4.69±0.13 ^##^ |
| Ventral Tegmental Area | 3.96±0.22 | 4.19±0.23 | 4.50±0.15 ^##^,^+^ |
| Zona Incerta | 4.47±0.27 | 4.75±0.30 | 5.08±0.20 ^##^ |
| Medulla | 55.30±3.10 | 60.60±4.00 * | 65.41±2.04 ^###^,^+^ |
| Cerebellum | 155.98±11.33 | 163.18±9.32 | 176.63±7.69 ^#^,^+^ |
