# Supplementary material for: Volumes of brain structures in captive wild-type and laboratory rats: 7T magnetic resonance in vivo automatic atlas-based study
Source: PLoS One. 2019 Apr 11;14(4):e0215348. doi: 10.1371/journal.pone.0215348 (PMC6459519; doi:10.1371/journal.pone.0215348)
Supplement: S2 Table — (DOCX) [file pone.0215348.s002.docx]

**S2 Table.** Validation of the automatic segmentation. Individual non-normalized volumes of the hippocampi calculated automatically or manually. The last column presents volume difference between these two methods of segmentation (VD).

| **Strain/Group** | **Results of automatic segmentation of the hippocampus V_auto_ [mm3]** | **Results of the manual segmentation of the hippocampus V_manual_ [mm3]** | **Percentage volume difference VD [%]** |
| --- | --- | --- | --- |
| WWCPS | 113.04 | 111.53 | 1.34 |
| WWCPS | 108.51 | 100.26 | 7.90 |
| WWCPS | 110.97 | 106.66 | 3.97 |
| WWCPS | 112.43 | 110.85 | 1.41 |
| WWCPS | 123.40 | 113.70 | 8.19 |
| WWCPS | 106.72 | 97.77 | 8.75 |
| WWCPS | 114.13 | 108.03 | 5.49 |
| WWCPS | 108.24 | 106.66 | 1.47 |
| BN | 128.69 | 123.63 | 4.01 |
| BN | 117.40 | 114.13 | 2.83 |
| BN | 119.63 | 122.49 | 2.36 |
| BN | 123.11 | 121.05 | 1.69 |
| BN | 127.08 | 113.05 | 11.69 |
| BN | 123.75 | 118.36 | 4.46 |
| BN | 117.09 | 103.23 | 12.58 |
| BN | 131.50 | 120.57 | 8.67 |
| WISTAR | 136.09 | 130.85 | 3.93 |
| WISTAR | 131.91 | 126.32 | 4.33 |
| WISTAR | 135.09 | 120.79 | 11.18 |
| WISTAR | 134.73 | 135.75 | 0.76 |
| WISTAR | 126.55 | 125.38 | 0.93 |
| WISTAR | 135.01 | 133.56 | 1.08 |
| WISTAR | 132.23 | 122.81 | 7.39 |
| WISTAR | 135.05 | 124.60 | 8.05 |
