# Supplementary material for: Volumes of brain structures in captive wild-type and laboratory rats: 7T magnetic resonance in vivo automatic atlas-based study
Source: PLoS One. 2019 Apr 11;14(4):e0215348. doi: 10.1371/journal.pone.0215348 (PMC6459519; doi:10.1371/journal.pone.0215348)
Supplement: S3 Table — (DOCX) [file pone.0215348.s003.docx]

**S3 Table.** Coefficient of variance [%] for different normalized volumes of the brain structures in WWCPS, BN and Wistar rats. *p<0.05 WWCPS vs. BN, ^##^p<0.01 WWCPS vs. Wistar, ^+^p<0.05 BN vs. Wistar.

| **Structures** | **WWCPS** | **BN** | **Wistar** | **p** |
| --- | --- | --- | --- | --- |
| ***Brain (scaled)*** | 5.00 | 4.00 | 3.26 | ^+^ |
| ***Cortex*** | 0.80 | 1.16 | 0.67 |  |
| Auditory Cortex | 1.13 | 1.02 | 0.92 |  |
| Cingulate Cortex | 1.29 | 1.69 | 1.43 |  |
| Entorhinal Cortex | 1.22 | 1.46 | 1.03 |  |
| Frontal Cortex Association | 1.20 | 0.92 | 0.63 |  |
| Insular Cortex | 1.02 | 1.36 | 0.93 |  |
| Medial Prefrontal Cortex | 0.85 | 1.04 | 0.94 |  |
| Motor Cortex | 1.16 | 2.07 | 1.83 |  |
| Orbitofrontal Cortex | 0.83 | 1.80 | 1.36 |  |
| Parietal Cortex Association | 1.01 | 1.21 | 0.63 |  |
| Piriform Cortex | 1.15 | 1.33 | 1.00 |  |
| Retrosplenial Cortex | 2.17 | 2.69 | 1.29 | ^#^ |
| Somatosensory Cortex | 1.57 | 1.74 | 2.13 |  |
| Temporal Cortex Association | 2.24 | 1.60 | 0.85 |  |
| Visual Cortex | 4.73 | 2.46 | 1.04 |  |
| ***Hippocampus*** | 1.26 | 1.09 | 1.27 |  |
| Antero Dorsal | 2.79 | 1.94 | 1.37 |  |
| Posterior | 1.28 | 2.34 | 1.71 |  |
| Postero Dorsal | 1.48 | 1.63 | 1.32 |  |
| Subiculum | 2.71 | 2.22 | 2.64 |  |
| Ventral | 2.17 | 1.25 | 1.47 |  |
| ***Thalamus*** | | | | |
| Dorsolateral | 1.99 | 1.38 | 1.75 |  |
| Midline Dorsal | 2.71 | 2.20 | 3.39 |  |
| Ventromedial | 2.42 | 1.94 | 3.04 |  |
| ***Hypothalamus*** | | | | |
| Lateral | 1.97 | 3.11 | 1.82 |  |
| Medial | 2.22 | 5.68 | 2.37 |  |
| ***Accumbens*** | | | | |
| Core | 1.05 | 0.97 | 0.46 |  |
| Shell | 0.93 | 0.88 | 0.36 | ^##^, ^+^ |
| ***Other structures*** | | | | |
| Amygdala | 1.49 | 1.32 | 1.41 |  |
| Bed Nucleus Stria Terminalis | 1.07 | 0.57 | 0.42 | *, ^##^ |
| Caudate Putamen | 2.20 | 1.75 | 1.92 |  |
| Corpus Collosum | 0.76 | 1.34 | 2.29 | ^##^ |
| Diagonal Band | 0.87 | 2.28 | 1.10 |  |
| Globus Pallidus | 1.22 | 1.39 | 1.72 |  |
| Internal Capsule | 1.58 | 1.44 | 1.14 |  |
| IPAC | 1.36 | 2.22 | 1.79 |  |
| Medial Geniculate | 1.27 | 2.10 | 1.83 |  |
| Mesencephalic Region | 1.74 | 3.27 | 3.07 | ^#^ |
| Olfactory Nuclei | 1.41 | 1.67 | 1.36 |  |
| Olfactory Tubercle | 1.47 | 1.75 | 1.63 |  |
| Periaqueductal Grey | 2.30 | 2.82 | 2.41 |  |
| Pons | 3.85 | 11.62 | 3.61 |  |
| Raphe | 2.03 | 2.30 | 1.34 | ^#^ |
| Septum | 3.63 | 2.55 | 4.23 |  |
| Substantia Innominata | 1.15 | 1.61 | 1.35 |  |
| Substantia Nigra | 1.85 | 2.02 | 1.75 |  |
| Superior Colliculus | 1.69 | 1.87 | 1.83 |  |
| Ventral Pallidum | 1.79 | 2.87 | 1.62 |  |
| Ventral Tegmental Area | 1.61 | 1.70 | 1.06 |  |
| Zona Incerta | 1.73 | 2.82 | 0.96 |  |
| Medulla | 2.39 | 3.52 | 2.05 |  |
| Cerebellum | 2.77 | 2.52 | 1.60 |  |
