# Supplementary material for: Volumes of brain structures in captive wild-type and laboratory rats: 7T magnetic resonance in vivo automatic atlas-based study
Source: PLoS One. 2019 Apr 11;14(4):e0215348. doi: 10.1371/journal.pone.0215348 (PMC6459519; doi:10.1371/journal.pone.0215348)

S1 Figure. Visualization of the manual segmentation of the hippocampus (A-C) and the olfactory bulbs (D-F) in WWCPS (A, D), BN (B, E) and Wistar rats (C, F).

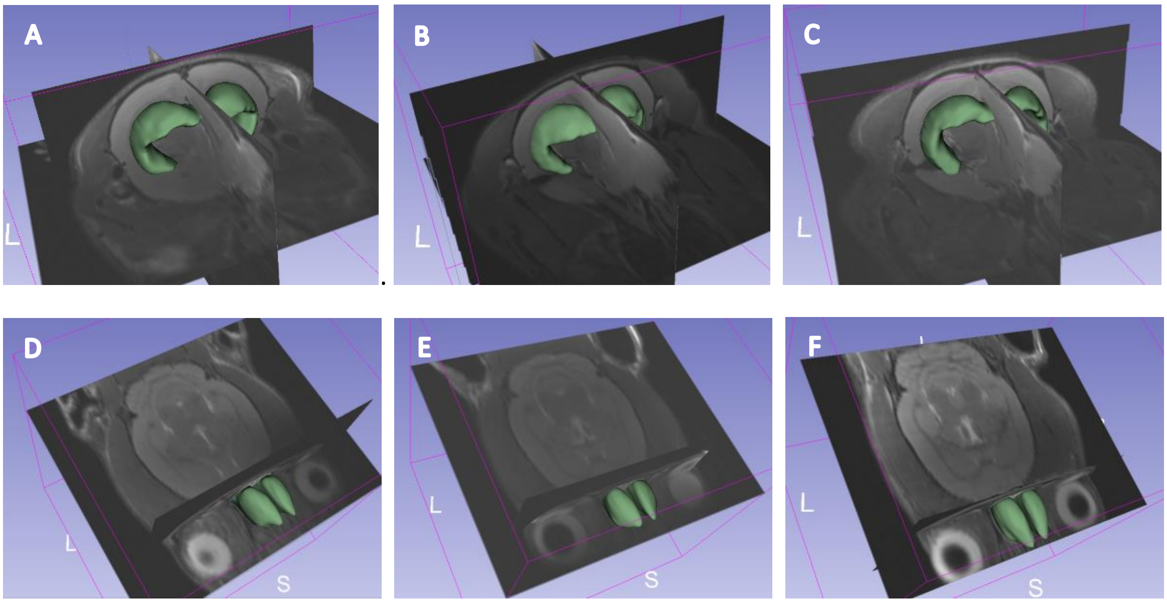

Supplement: S1 Fig — (PDF) [file pone.0215348.s006.pdf]
